# Glycogen synthase kinase 3, circadian rhythms, and bipolar disorder: a molecular link in the therapeutic action of lithium

**DOI:** 10.1186/1740-3391-5-3

**Published:** 2007-02-12

**Authors:** Sevag A Kaladchibachi, Brad Doble, Norman Anthopoulos, James R Woodgett, Armen S Manoukian

**Affiliations:** 1 Division of Signaling Biology, Ontario Cancer Institute, University Health Network, 610 University Avenue, Toronto, Ont. M5G 2M9, Canada; 2 Stem Cell and Cancer Research Institute, McMaster University, 1200 Main Street West, Hamilton, Ont. L8N 3Z5, Canada; 3 Samuel Lunenfeld Research Institute, 600 University Avenue, Toronto, Ont. M5G 1X5, Canada

## Abstract

**Background:**

Bipolar disorder (BPD) is a widespread condition characterized by recurring states of mania and depression. Lithium, a direct inhibitor of glycogen synthase kinase 3 (GSK3) activity, and a mainstay in BPD therapeutics, has been proposed to target GSK3 as a mechanism of mood stabilization. In addition to mood imbalances, patients with BPD often suffer from circadian disturbances. GSK3, an essential kinase with widespread roles in development, cell survival, and metabolism has been demonstrated to be an essential component of the *Drosophila *circadian clock. We sought to investigate the role of GSK3 in the mammalian clock mechanism, as a possible mediator of lithium's therapeutic effects.

**Methods:**

GSK3 activity was decreased in mouse embryonic fibroblasts (MEFs) genetically and pharmacologically, and changes in the cyclical expression of core clock genes – *mPer2 *in particular – were examined.

**Results:**

We demonstrate that genetic depletion of GSK3 in synchronized oscillating MEFs results in a significant delay in the periodicity of the endogenous clock mechanism, particularly in the cycling period of *mPer2*. Furthermore, we demonstrate that pharmacological inhibition of GSK3 activity by kenpaullone, a known antagonist of GSK3 activity, as well as by lithium, a direct inhibitor of GSK3 and the most common treatment for BPD, induces a phase delay in *mPer2 *transcription that resembles the effect observed with GSK3 knockdown.

**Conclusion:**

These results confirm GSK3 as a plausible target of lithium action in BPD therapeutics, and suggest the circadian clock mechanism as a significant modulator of lithium's clinical benefits.

## Background

Bipolar disorder is a common, chronic, severe, and often life-threatening illness, characterized by recurrent episodes of two diametrically opposite mood states: mania and depression [1]. Despite clinical efforts spanning four decades, the pathophysiology and etiology of BPD remain unclear, in part due to the inherent genetic heterogeneity of affective mood disorders [2]. Current consolidated models for BPD suggest that interplay between genetic, epigenetic, and environmental factors cause the disease [1,3].

Modes of therapy deemed to be effective in alleviating symptoms have been puzzling with respect to their modes of action at the molecular, cellular, systemic and behavioural levels. Despite four decades of clinical use, and demonstrated efficacy in reducing the frequency and severity of recurrent affective episodes, the molecular mechanisms underlying the therapeutic actions of lithium, a hallmark of BPD therapeutics, remain to be fully elucidated [4,5]. The identification of the molecular target(s) of mood stabilizers would not only facilitate the development of improved modes of treatment, but also provide a basis for the delineation of the pathophysiology of affective mood disorders.

Features of circadian rhythmicity represent an endophenotype that has received a significant amount of attention [2,6]. The cyclic nature of bipolar disorder itself, and clinical features of depression such as premature early morning awakening from sleep and diurnal variation in mood, shortened REM sleep latency, advances in hormonal and temperature rhythms, as well as activity, highlight the prevalence of circadian rhythm abnormalities in affected subjects, with particular tendencies towards phase advances and/or shortened periods [2,6-8]. It is therefore of great consequence that lithium has been found to modify the free running period or phase of numerous synchronized behavioural, physiological, and biochemical rhythms in various experimental settings conducted on a phylogenetically diverse variety of organisms, including humans [9]. Most consistent among these modifications is the effect of period lengthening and/or phase delay. Examples of these lithium-modified rhythms include delayed rhythms of drinking and locomotor activity in rodents [10,11]; as well as temperature, activity, REM sleep latency and sleep/wake rhythms of human subjects [12-14]. Moreover, lithium-induced period lengthening has also been demonstrated on the pacemaking properties of single cells, suggesting a direct modulation of the clock mechanism [15].

GSK3, a serine/threonine kinase encoded in mammals by two isoforms, *GSK3α *and *GSK3β*, is a direct, in vitro and in vivo target of inhibition by lithium [16-18]. *Shaggy *(*Sgg*), the *Drosophila *homologue of *GSK3*, plays a central role in determining circadian period length in flies [19], providing a compelling molecular target for the mechanistic basis of lithium's therapeutic effects. The circadian system functions to promote the optimal temporal organization of a variety of specialized states, segregating in time those which are mutually incompatible, thus integrating neurotransmitter, endocrine and behavioural mechanisms whose dysregulation may be involved in the pathophysiology of affective disorders [6]. While there are many differences in the molecular and genetic details of the circadian machinery in mammals and *Drosophila*, the basic regulatory principles are maintained [20,21]. Central to the molecular circadian circuitry of various phylogenetically diverse organisms are the interconnected positive and negative autoregulatory feedback loops of transcription and translation, protein-protein interaction, phosphorylation, nuclear translocation, and degradation, whose imposed delays combine to create a molecular cycle that approximates the ~24 hr environmental LD period [21]. A significant determinant of period length is the delay in the nuclear translocation of negative regulatory complexes, the timing of which is tightly regulated [22-27]. In *Drosophila*, SGG was found to modulate the nuclear localization of the TIMELESS/PERIOD inhibitory complex, through its phosphorylation of TIM protein, promoting nuclear translocation of the PER/TIM complex [19]. As such, overexpression of SGG resulted in a shortening of the intrinsic period, whereas a reduction of SGG activity had a period lengthening effect, precisely the effect attributed to lithium on the free-running period of most organisms studied, including *Drosophila *[28].

The evolutionarily conserved nature of both the circadian molecular mechanism [21,29] and the period altering effects of lithium led to the present investigation of the role of GSK3 in the mammalian circadian clock. Examination of the transcriptional profiles of core clock genes in synchronized mouse embryonic fibroblasts (MEFs), following genetic or pharmacological reduction of GSK3 activity, revealed a consistent period lengthening/phase delaying effect. Taken together, these results provide a molecular basis for the therapeutic efficacy of lithium, and validate GSK3 as a candidate mammalian "core" clock gene.

## Methods

### Generation of stable mouse embryonic fibroblast lines with *GSK3α *knockdown in a *GSK3β *nullizygous background

An siRNA target sequence, 5'-aaagcgtcagtcggggctatg-3', located in the coding sequence of the N-terminal extension unique to *GSK3α *was identified using Ambion's siRNA target finder [30]. Through BLAST analysis, this sequence was determined to be unique to mouse *GSK3α*. The Silencer™ Express siRNA expression cassette (SEC) kit (Ambion) was used to create a SEC for *GSK3α *with oligos directed against the *GSK3α *target sequence described above. The SEC was cloned into pCR^®^-Blunt-II-TOPO^® ^(Invitrogen) and the sequence was verified. To allow for stable cell line generation, the SEC was sub-cloned into the unique EcoRI site of the plasmid pPUR (Clontech) to create the plasmid pPUR_ASEC1. Immortalized MEFs derived from *GSK3β *nullizygous embryos [31] were transfected with ScaI-linearized pPUR_ASEC1 using Lipofectamine 2000 (Invitrogen). Two days after transfection, the cells were put under selection using 2 μg/ml puromycin (InVivogen). Puromycin-resistant clones were isolated, expanded, and tested by western blotting to determine the level of GSK3α knockdown.

### Generation of GSK3β^(-/-)^;GSK3α^(flox/-) ^fibroblasts

E14K mouse embryonic stem cells with LoxP-recombination sites flanking exon 2 of GSK3 (both alleles), were made null for GSK3β through conventional gene targeting by first replacing exon 2 with a neo cassette for positive selection with G418 and then rendering the locus homozygous through selection in 2.5 mg/ml G418 as previously described [32]. These GSK3β^(-/-)^;GSK3α^(flox/flox) ^ES cells were injected into blastocysts derived from B6 mice to generate chimeric embryos. A total of 14 embryos from 3 pregnant recipient mothers were harvested at day 16.5 dpc to generate separate cultures of primary mouse fibroblasts from each embryo. To select MEFs derived from the injected ES cells, cultures were placed under selection with 1 mg/ml G418. Only 1 of the 14 pups (pup 10) gave rise to a large number of G418-resistant MEFs, while the others yielded none. The resistant MEFs were passaged 10 times, maintaining selection in G418 and using a split ratio of 1:3. After 10 passages, the cells entered crisis. Immortalized fibroblasts that escaped crisis were transduced with a retrovirus expressing self-excising cre recombinase to generate the GSK3β^(-/-)^; GSK3α^(flox/-) ^(3/4KO) fibroblast lines used in this study [33].

### Cell culture and serum shock procedures

MEFs were grown in Dulbecco's modified eagle medium (DMEM) supplemented with 5% fetal bovine serum (GIBCO) and a mixture of penicillin-streptomycin-glutamine (PSG from GIBCO). Puromycin-resistant MEF lines were grown, maintained, serum shocked, and serum starved in medium containing 2 μg/mL puromycin. Where indicated, serum shock and serum starvation media were supplemented with a final concentration of 20 mM lithium Chloride or 25 μM kenpaullone (Calbiochem). The serum shock was performed as described previously by Balsalobre and colleagues [34], with slight modifications: 4 × 10^5 ^cells/10 cm Petri dish were plated 6–7 days before the experiment, and serum shocked for 2 hours at TP0, and thereafter serum starved in DMEM for the duration of the experiment. At the indicated times, following two ice-cold 10 ml PBS washes, the cells were lysed in 1 mL of RLT buffer (QIAGEN) for RNA harvests, or 1 mL of RIPA buffer for protein harvests. The lysates were collected, flash frozen in an ethanol/dry ice bath, and stored at -70°C until extraction of whole cell RNA or SDS-PAGE sample preparation.

### RNA purification and cDNA synthesis

All harvested RLT lysates were homogenized using QIAshredder™ columns (QIAGEN), and RNA was extracted from the homogenized lysates using RNeasy^® ^columns (QIAGEN). RNA concentrations were measured using standard UV spectrophotometry at 260 nm. All harvested samples were diluted down to the lowest registered concentration, and electrophoresed through a denaturing gel to verify RNA quality. 1 μg of total RNA was reverse-transcribed with Stratascript™ RT (Stratagene) at 42°C for 1 hr in a final reaction volume of 10 μl, and subsequently diluted 5-fold to generate a poly-dT cDNA library of each harvested sample, for use as template in PCR amplification.

### PCR analysis of transcriptional profiles

For all transcriptional profiles, PCR reactions for all time-points were prepared simultaneously, where 5 μl of cDNA was added to 45 μl of PCR mixture [10× reaction buffer, 0.5 unit Taq DNA polymerase (Roche), 0.2 mM dNTPs, and 50 pmol of primers]. PCR cycles were as follows: 95°C for 1 min, cycles of 45 s at 95°C, 60 s at 60°C, and 120 s at 72°C, and a final extension period of 10 min at 72°C. Amplification consisted of 25 cycles for *GAPDH*, 27 cycles for *RevErbα *and *Bmal1*, 30 cycles for *mCry1*, and 34 cycles for *mPer2*. The following forward and reverse primers were designed, synthesized (ACTG Corp.), and utilized in the above reactions: *GAPDH *forward 5'-GGTGAAGGTCGGTGTGAACGGATTTGGCCG-3', *GAPDH *reverse 5'-CTCCTTGGAGGCCATGTAGGCCATGAGGTC-3'; *RevErbα *forward 5'-CAGCTTCCAGTCCCTGACTCAAGGTTGTCCCACATAC-3', *RevErbα *reverse 5'-GGCGTAGACCATTCAGCGCTTCATTATGACGCTGAG-3'; *Bmal1 *forward 5'-CCGTGCTAAGGATGGCTGTTCAGCACATG-3', *Bmal1 *reverse 5'-GTCCTCTTTGGGCCACCTTCTCCAGAGGG-3'; *mCry1 *forward 5'-GTGAACGCCGTGCACTGGTTCCGAAAGGGAC-3', *mCry1 *reverse 5'-GTCATGATGGCGTCAATCCACGGGAAGCCTG-3'; *mPer2 *forward 5'-GATCAGCTGCCTGGACAGTGTCATCAGGTACC-3', and *mPer2 *reverse 5'-CTGAGCGTCGAGGTCCGACTAGGGAACTCAGCC-3'. PCR amplification with samples from individual serum shock experiments was carried out at least twice to insure proper replication of resulting transcriptional profiles.

### Western Blot analysis

MEFs were rinsed 3× with PBS, placed on ice and then scraped into either ice-cold hypotonic lysis buffer (50 mM Tris pH 7.4, 1 mM EDTA, 50 mM Tris pH 7.4) supplemented with a protease inhibitor cocktail (Roche) (200 μl/well of 6-well dish) in the case of the clonal selection analyses, or 1 ml of ice-cold RIPA buffer [1% (v/v) NP-40, 1% (w/v) sodium deoxycholate, 0.1% (w/v) SDS, 150 mM NaCl, 10 mM sodium phosphate (pH 7.2), 2 mM EDTA, 50 mM NaF, 1 mM benzamidine, 25 mM β-glycerophosphate] supplemented with a protease inhibitor cocktail (Roche) for serum shocked MEFs. Hypotonic lysates were centrifuged for 1 hour at 4°C at 16,100 × g. RIPA lysates were homogenized with 23 gauge needle syringes, and centrifuged at 13,500 × g for 12 minutes at 4°C in an Eppendorf microcentrifuge. The clarified extract was collected carefully so that the pellet and any floating cellular debris were not aspirated during pipeting. Protein content of the lysates was determined using the Dc Protein Assay (BioRad). Samples were run through 8% SDS polyacrylamide gels and electrophoretically transferred to PVDF membranes. The blots were blocked for 1 hour (RT) in 5% skim milk powder/TBS and then incubated for 1 hour (RT) with the following mouse monoclonal primary antibodies diluted in 2% skim milk powder/TBST (TBS + 0.1% Tween-20): GAPDH (1:100 000, clone 6C5, Abcam); β-Actin (1:20 000, clone AC-15, Abcam); β-catenin (1:1000, clone 14, BD Transduction Labs); and GSK-3 (1:1000, clone 4G-1E, Upstate). Blots were rinsed 3× with the antibody diluent and were then incubated for 1 hour (RT) with goat anti-mouse secondary antibody conjugated to HRP (BioRad) diluted 1:10 000 in the same buffer. After 5 washes with TBST (no milk), blots were incubated for 5 minutes with SuperSignal West Pico chemiluminescent substrate (Pierce). X-Omat blue scientific imaging film (Kodak) was used to detect the chemiluminescent signal.

## Results

### Phase relationship of core clock genes in wild-type MEFs

Investigation of the role and/or requirement of GSK3 in the circadian clock mechanism is hampered by the fact that *GSK3β *nullizygous embryos develop normally to mid-gestation, but die around day 14 of embryonic development [31]. However, the discovery by Balsalobre and colleagues that high concentrations of serum can synchronize the oscillation of clock genes in Rat-1 fibroblasts established the existence of peripheral clocks [34], whose oscillatory mechanism is similar and comparable to the central oscillator residing in the suprachiasmatic nuclei (SCN) of the hypothalamus [23,35].

**Figure 1:**
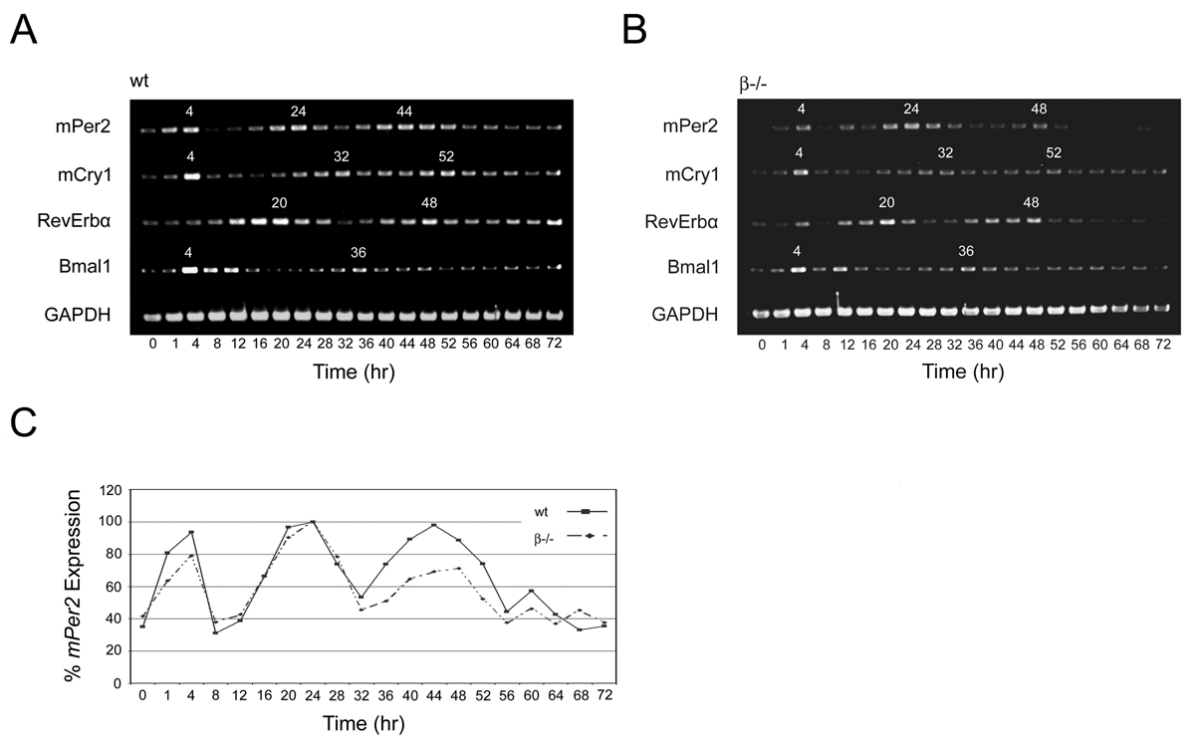
**Circadian oscillation profiles of clock genes in wild-type and *GSK3β*^**-/- **^MEFs**. **A**, wild type and **B**, *GSK3β*^-/- ^cells were synchronized, harvested, processed, and the gene products were amplified as described in the Materials and Methods. The resulting transcriptional profiles of murine *GAPDH*, m*Per2*, m*Cry1*, *RevErbα*, and *Bmal1 *were analyzed by reverse-transcription PCR. The subjective time points (TP) of peak expression are designated in white above the corresponding bands for each transcript examined. Panel **C **is a graphical depiction of m*Per2 *transcriptional oscillation based on relative values derived from densitometric measurements of PCR-amplified DNA bands in panels **A **and **B **expressed as percentages of the highest recorded value in each respective data set.

Although the expression patterns of known core clock genes have been previously documented, the control phase relationships among selected clock components were established in wild-type MEFs for comparative purposes prior to the investigation of the effects of *GSK3 *deficiency on the clock mechanism. Following serum shock treatment, the oscillatory transcriptional expression profile of four clock genes – *mPer2 *(*Period 2*), *mCry1 *(*Cryptochrome 1*), *Bmal1 *and *RevErbα*, were examined by reverse transcription (RT)-PCR. The *Clk *(Clock) gene was excluded from this study as *Clk *mRNA and CLK protein are constitutively expressed [36]. The resulting expression profiles (Fig. 1A) and phase relationships of these four genes were consistent with previously published accounts [23,24,34,37,38].

### Phase relationship of core clock genes in *GSK3β*-null MEFs

Having established the oscillatory phases of *mPer2*, *mCry1*, *RevErbα*, and *Bmal1 *in wild-type MEFs, the effects of *GSK3β *deficiency on circadian rhythmicity were investigated in *GSK3β *nullizygous MEFs [31]. For *mCry1*, *RevErbα*, and *Bmal1*, peak times of transcription were identical to those observed in their wild type counterparts. The only noticeable alteration observed was a discrete shift in the *mPer2 *peak occurring in the second cycle, which is ~TP44 in wild type MEFs (Fig. 1A,C), and delayed to TP48 in *GSK3β*^-/- ^MEFs (Fig. 1B,C), while the first peak occurs at TP24 in both genotypes. In a setting where the circadian clock mechanism, within a functional range, is relatively insulated from reduction in absolute levels of GSK3, the absence of GSK3β could be compensated by the actions of GSK3α, where in a 72 hr time span, a 50% reduction in GSK3 levels may still be sufficient to fully carry out the enzyme's functional duties. Therefore, the targeted "knock down" of the *GSK3α *isoform in the *β*-null MEFs was undertaken in order to minimize the contribution of GSK3 in the generation of transcriptional oscillations.

### GSK3α RNAi knockdown in a GSK3β nullizygous background lengthens the *mPer2 *transcriptional period

**Figure 2:**
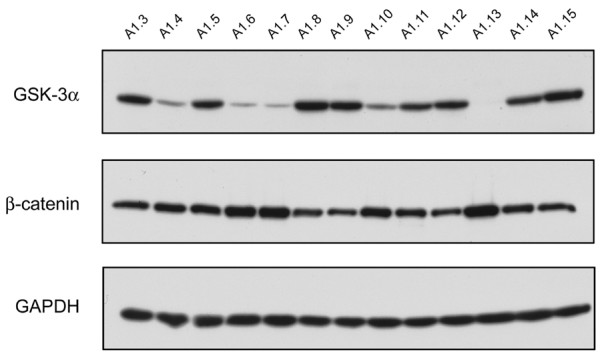
**Analysis of *GSK3α *knockdown efficacy in a *GSK3β *nullizygous background**. Puromycin-resistant, *GSK3α*-knockdown MEF lines were generated from *GSK3β*^-/- ^MEFs as described in the Materials and Methods. A total of 13 individual clones (A1.3-A1.15) were subsequently isolated, expanded, and tested by Western blotting to determine the level of GSK3α knockdown, as well as any concomitant increase in cytosolic levels of β-Catenin protein. GAPDH levels were used as a loading control.

A total of 13 puromycin-resistant clones were isolated (A1.3 – A1.15), expanded, and tested by western blotting to determine the level of *GSK3α *knockdown. Initial western blot analysis of the stable *GSK3α *knockdown clones was done as soon as there were a sufficient number of cells (Fig. 2). The levels of GSK3α were assessed, as well as the cytosolic levels of β-catenin protein, which should increase as total GSK3 levels decrease [39-41]. A clear reduction in GSK3α protein levels with a concomitant increase in β-catenin levels was detected in several of the puromycin resistant clones, namely A1.4, 6, 7 and 13 (Fig. 2). In particular, the A1.13 clone had barely detectable GSK3α protein as well as the expected massive increase in cytosolic β-catenin levels. However, re-analysis of these four clones, after only 3 additional passages, revealed that despite retention of puromycin resistance, clone 13 had recovered GSK3α expression at a level comparable to wild type, while clone 7 had partially (~50% of wild-type) recovered GSK3α expression (data not shown). The A1.4 and A1.6 clones initially identified as having a sub-maximal, but significant level of knockdown still retained the same degree of reduction, with A1.6 displaying a higher level of GSK3α knockdown than A1.4. The complete "reversion" of clone 13 suggested that there may be selection against cells with extremely low GSK3 levels and that GSK3 may be essential for MEF viability. The A1.4 and A1.6 clones were selected for subsequent serum-shock analysis. In order to ensure that any potential circadian effects observed in these clones were not an artefact of the transfection process, the fully reverted A1.13 clone was serum shocked and analyzed. The transcriptional profile of *mPer2*, *mCry1*, *Bmal1 *and *RevErbα *in the A1.13 MEFs were found to be identical to those observed in the parental *GSK3β*-null MEFs (data not shown).

**Figure 3:**
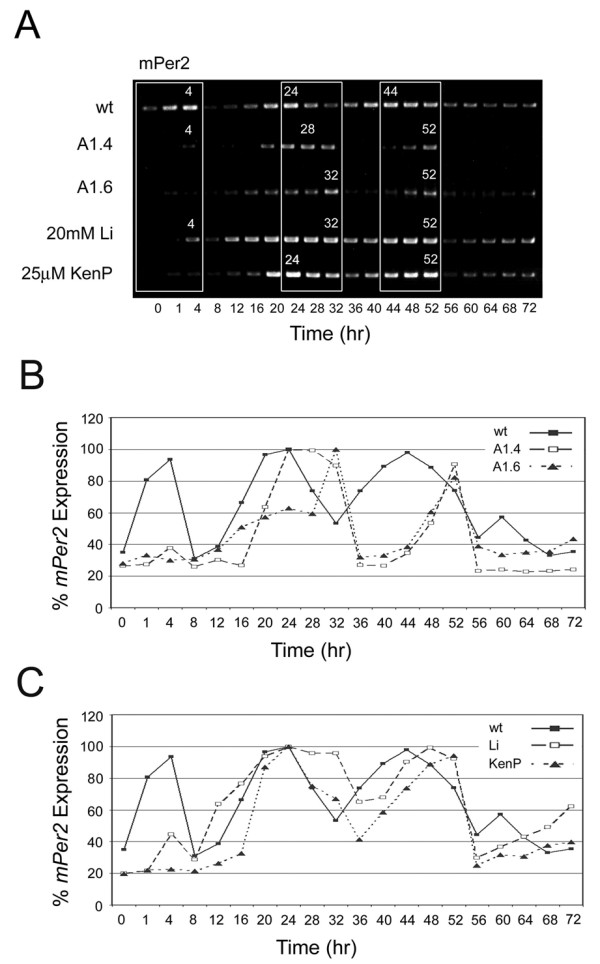
**Circadian oscillation profiles of *mPer2 *following pharmacological or genetic GSK3 inhibition**. The transcriptional profile *mPer2 *(**A**) was analyzed by reverse-transcription PCR in: wild-type, *GSK3β*^-/-^/*GSK3α*^RNAi ^(clones A1.4 and A1.6); as well as 20 mM Lithium or 25 μM kenpaullone treatment in a wild-type background. The subjective time points of peak expression are designated in white above the corresponding bands for each transcript examined. The time intervals where these effects are most visible (TP0-4, TP24-32, and TP44-52) are isolated in white boxes. The effects of genetic (**B**) and pharmacological (**C**) interference of GSK3 activity on *mPer2 *transcriptional oscillation are graphically depicted based on relative values derived from densitometric measurements of PCR-amplified DNA bands in panel **A **expressed as percentages of the highest recorded value in each respective data set.

**Figure 4:**
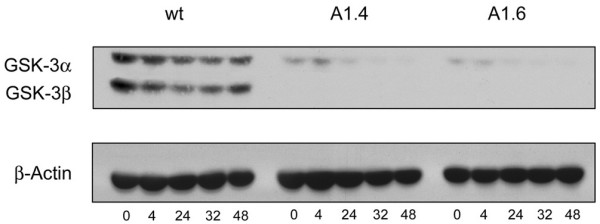
**Analysis of total GSK3 expression prior to and following serum-shock**. To verify that effects observed at the level of transcription in clones A1.4 and A1.6 corresponded to an expected level of GSK3 knockdown, at the indicated time points, protein and RNA samples were simultaneously isolated from harvested wild-type, A1.4, and A1.6 cells in order to monitor levels of GSK3 expression prior to and following serum shock. RNA samples were subsequently used for reverse-transcription PCR analysis (as seen in Fig. 3), while the protein samples were subjected to Western blot analysis. Protein samples were harvested at TP0, TP4, TP24, TP32 and TP48, and blotted for total GSK3. β-Actin levels were used as a loading control.

The clock gene mRNA expression profiles were examined in the A1.4 and A1.6 clones. Whereas the lengthening in the period of *mPer2 *observed in the parental *GSK3β*-null cell line was subtle in the context of this assay, a much more distinctive and exaggerated *mPer2 *period lengthening phenotype emerged from the additional RNAi-mediated reduction of GSK3α. The effect on *mPer2 *was most pronounced in A1.6, which (1) lacked the serum-induced transcriptional peak at TP4, and (2) had a dramatic ~8 hr delay in transcriptional repression, with peaks of transcription occurring at TP32 and TP52 (Fig. 3A,B). In parallel with the A1.4 and A1.6 RNA samples, protein extracts were harvested at 0, 4, 24, 36 and 48 hours following the serum shock, in order to monitor the relative reduction in levels of GSK3 expression in the two clones (Fig. 4). As expected, based on the screening process, the A1.6 clone achieved the highest level of "knock-down", especially prior to (TP0) and immediately following (TP4) serum shock, time points at which A1.4 expressed slightly higher levels of GSK3 (Fig. 4). These observations were consistent with the initially intermediate effect on *mPer2 *transcription seen with the A1.4 clone, which had an abrogated but detectable serum-induced activation of *mPer2 *expression at TP4, and a ~4 hr delay in transcriptional repression during the first cycle, with a peak at ~TP28 (Fig. 3A,B). However, by TP48, at which A1.4 and A1.6 both have comparable levels of residual GSK3 expression, a delay of ~8 hrs is observed with both clones, with a peak occurring at TP52 (Fig. 3A,B).

### Lithium lengthens *mPer2 *period and phenocopies *GSK3 *knockdown

Lithium, a direct GSK3 inhibitor [16-18], is known to consistently lengthen the circadian period of a variety of organisms in a dose-dependent manner [15,42,43]. In fact, lithium has been shown to lengthen the period of firing rate in individual neurons of the SCN [15], suggesting an effect on the pacemaking properties of single cells, in addition to overt effects at the behavioural level. The mechanistic nature of this effect, however, at the level of the molecular circadian machinery, still remains to be elucidated. Having demonstrated a period lengthening effect in *GSK3 *knockdown MEFs, specifically with respect to *mPer2 *transcriptional oscillation, the molecular effect of lithium on period length was investigated in wild type MEFs. A final lithium concentration of 20 mM was chosen representing the upper range of GSK3 inhibition, based on the in vitro dose-response curve of GSK3 to lithium, as described with respect to Tau as well as RevErbα [17,44,45].

As can be seen in the transcriptional profiles, lithium was also found to produce a similar period lengthening effect. For *mPer2*, peaks of transcription were delayed by 4–8 hrs to ~TP32 and TP52 (Fig. 3A,C), but unlike A1.6, a modest induction of *mPer2 *was achieved at TP4, likely representing a serum response initiated prior to the full inhibitory effect of lithium, whose uptake is known to be maximal at ~2 hr in human fibroblasts [46].

### Selective inhibition of GSK3 by kenpaullone lengthens *mPer2 *period

The paullones were first reported as ATP-competitive inhibitors of CDK1/cyclin B [47], and subsequently shown to inhibit GSK3 and other CDKs [48], and therefore represent an alternative class of GSK3 inhibitors that are structurally and mechanistically unrelated to lithium. Although Alsterpaullone is a more potent inhibitor of GSK3 than kenpaullone, it has been shown to be less specific and therefore less suitable for cell-based assays [49]. Furthermore, continuous exposure of MEFs to Alsterpaullone for a period of 72 hr proved to be toxic, as most plated cells perished after "only" ~32 hrs (data not shown). Also, a relatively high initial concentration of kenpaullone was administered to account for likely degradation of the compound over a 72 hr period. As such, kenpaullone, at a final concentration of 25 μM, was administered during and immediately following serum shock and its effect on clock gene transcription was investigated. Analysis of the subsequent transcriptional profiles revealed a tangible effect on *mPer2 *period length. The serum-induced *mPer2 *transcriptional peak at TP4 was relatively muted (Fig. 3A,C), an effect seen with A1.4, A1.6, and lithium. In the first cycle, a normal subjective peak was observed at TP24, prior to a delayed trough at TP36, while the second peak of transcription was delayed to TP52 (Fig. 3A,C), again, as in both *GSK3*-knockdown clones and lithium. The effect of kenpaullone on GSK3 activity has been shown to be slow and gradual in various cell lines [50], and may account, at least in part, for the latency observed herein.

### Stable genetic ablation of GSK3α^(flox/-) ^in a GSK3β nullizygous background lengthens the *mPer2 *transcriptional period

**Figure 5:**
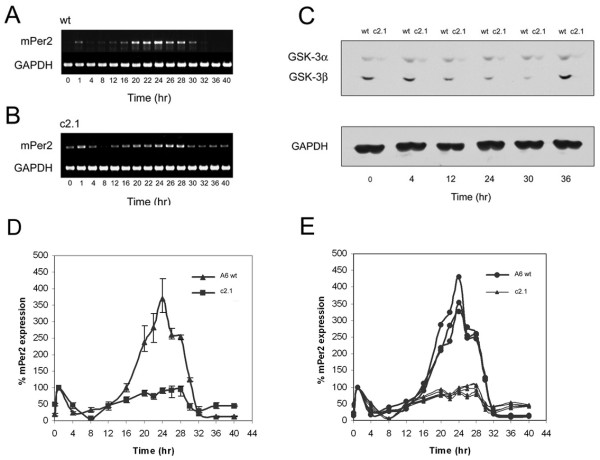
**Circadian oscillation profile of *mPer2 *in wildtype and GSK3β^(-/-)^; GSK3α^(flox/-) ^MEFs**. The transcriptional profile of *mPer2 *and *GAPDH *were analyzed by reverse-transcription PCR in the A6(wt) and c2.1(3/4 DKO) cell lines, as depicted in panels **A **and **B**, respectively. Protein samples harvested from whole-cell lysates in parallel to RNA samples harvested for transcriptional analysis at corresponding time points were analyzed by SDS-PAGE electrophoresis. Western blot analysis of these protein samples for total-GSK3 and GAPDH is depicted in Panel **C **for TPs 0, 4, 12, 24, 30, and 36. Panels **D **and **E **are graphical depictions of relative levels of *mPer2 *expression in A6 and c2.1 based on three separately harvested A6 RNA sample sets and six c2.1 RNA sample sets. Relative values derived from densitometric measurements of PCR-amplified DNA bands are expressed as percentage values of *mPer2 *at TP1.

In order to further confirm the circadian repercussions of in vitro GSK3 knockdown, and to attribute the observed effects on *mPer2 *transcriptional cycling specifically to GSK3 expression levels, a genetically defined stable knockout (3/4 DKO) MEF line was generated to circumvent issues of targeting and cellular response specificity presented by the RNAi method of GSK3 knockdown. Although numerous attempts at generating stable GSK3α/β double knockout MEFs from GSK3α^(flox/flox)^;GSK3β^(-/-) ^MEFs using cre-recombinase approaches have been unsuccessful to date, a GSK3α^(flox/-) ^stable MEF line, designated as c2.1, was successfully generated in a GSK3β nullizygous background. Using the earliest passage, and therefore youngest cell lines available, the *mPer2 *transcriptional profile was examined in both A6 (wildtype) MEFs, and c2.1 3/4 DKO MEFs over a 44 hr time span following serum shock (Fig. 5A,B). To monitor GSK3 expression in c2.1 relative to A6, protein extracts of both cell lines were harvested at 0, 4, 12, 24, 30 and 36 hours following the serum shock (Fig. 5C) in parallel with the serum shocked A6 and c2.1 RNA samples. Also, between TPs 20 and 32, RNA samples were harvested at 2 hr intervals, rather than 4 hr intervals, to better examine the transcriptional fluctuation of *mPer2 *in this critical time interval.

As previously described, *mPer2 *transcription in wildtype MEFs peaked at TP24, following the initial transcriptional response to serum shock at TP1 (Fig. 5A,D). By contrast, despite a similar peak in transcription at TP1, *mPer2 *transcriptional expression was blunted in amplitude and maintained near maximal levels between TP24 and TP28 and transcriptional repression in c2.1 was delayed until after TP28 (Fig. 5B,D), an effect consistent with previous results obtained by reducing GSK3 activity by shRNA, genetically or pharmacologically. To ensure reproducibility, A6 samples and transcriptional profiles were harvested and generated in triplicate, while the c2.1 samples were harvested and generated in sextuplicate (Fig. 5E).

The ~4 hr lengthening in *mPer2 *transcription in the c2.1 cell line, which is 75% deficient in GSK3 expression most resembles the ~4 hr *mPer2 *transcriptional lengthening observed with the previously characterized A1.4 line which exhibited a similar level of GSK3α expression.

## Discussion

Our results have demonstrated that both genetic and pharmacological reduction of GSK3 activity have a specific effect on the circadian transcriptional oscillation consisting of *mPer2 *period lengthening following serum shock-mediated in vitro synchronization, indicating a delay in phase. This is a particularly salient feature with respect to lithium's mood stabilizing properties, considering the correspondence between *mPer2 *expression and behavioural rhythms [51], suggesting that *mPer2 *may be more intimately involved in driven behaviour than other clock genes. The question now becomes: how does a decrease in GSK3 activity result in phase delay, specifically with respect to Per2, taking into consideration the current paradigm of the circadian clock mechanism? As the primary transcriptional inhibitors, period length is controlled by the mCRY proteins, through their regulated and mPER-heterodimerization-dependent timing of nuclear translocation [24,26,52]. This post-translational modification-imposed delay in nuclear translocation is optimized to maintain a period of ~24 hr [21,53]. Mathematical modeling of the mammalian circadian oscillator feedback loops has shown that extreme delays (~7 hr) can be achieved by varying the nuclear import rate of the PER/CRY complex, and delays >8.8 hr between *Per/Cry *mRNA and nuclear PER/CRY protein accumulation result in the abolishment of oscillation [54]. The predictions of this model were consistent with subsequent findings in cultured unsynchronized primary fibroblasts derived from *mPer2*^Luciferase-SV40 ^knockin mice, whose rhythmic periods were found to range from ~22 to ~30 hrs [55]. Furthermore, *mPer2 *expression was found to be phase delayed by 8 hr in *Clock *mutant mice compared to wild-type siblings [56]. Therefore, the maximal delay of ~8 hrs in *mPer2 *achieved in this present study further establishes this upper limit in period length allowed, beyond which catastrophic failure in rhythmicity may result. This level of periodic plasticity is likely allowed, at least in part, by the hallmark difference of ~8 hr between the transcriptional peaks of *mPer1/2 *and *mCry1 *[24], and the rate limiting role of mPER proteins for PER-CRY interaction and nuclear accumulation [52]. As such, assuming a PER/CRY complex nuclear translocation-promoting role of GSK3 similar to that of SGG and the PER/TIM complex in the *Drosophila *clock mechanism [19], it can be postulated that a reduction in the levels of GSK3 may produce a delay in the nuclear translocation of the PER/CRY complex. Most recently, Iitaka and colleagues [57] found that GSK3β (i) interacts with PER2 *in vitro *and *in vivo*, (ii) can phosphorylate PER2 *in vitro*, (iii) promotes the nuclear translocation of PER2 in COS1 cells and (iv) overexpression caused an ~2 hr advance in *mPer2 *phase. Extreme phenotypes are defined by mutations that alter period by >15% (3–4 hr) or lead to complete loss of circadian rhythms. In cases in which null mutants are lethal, an extreme phenotype can be deemed sufficiently compelling to define a clock gene of interest. The *mClk*, *Dbt*, *Tau*, and herein described *GSK3 *mutants fall into this category in which mutations caused >4 hr period changes and true null mutations are not available [58].

Lithium, a well documented direct inhibitor of GSK3, has been a cornerstone of BPD therapeutics despite a dearth of knowledge concerning the nature of its efficacy and its mode of action. Its ability to modulate circadian rhythmicity however, specifically its phase delaying properties in numerous, phylogenetically diverse organisms, has been a major factor in establishing a causal and symptomalogical link between BPD and circadian rhythms. In behavioural studies where concentrations of lithium comparable to those used in humans for the treatment of BPD (0.6–1.2 mM) are administered, the period altering effects are often measured over the span of weeks or months, and produce subtle but significant effects usually measured in minutes rather than hours [2]. Conversely, cell culture based assays with much more limited temporal windows of observation, such as the present study, have consistently demonstrated the need for higher concentrations of lithium [15,43,44,57,59]. In fact, clinical amelioration of mood in BPD patients often takes weeks to appear following chronic lithium administration [60], a time span consistent with a gradual realignment of the circadian clock. Furthermore, a number of studies have examined SNP mutations in the effective *GSK3β *promoter. Kwok and colleagues [61] showed that a T/C substitution in said promoter is associated with the level of transcriptional activity, with C substitution resulting in decreased expression, while three studies by Benedetti et al. have shown C/C homozygote BPD patients shared clinical features suggestive of a milder form of the illness, including a later age of onset, improved antidepressant response, and better long-term response to lithium mood stabilization [61-64]. The present results also offer a possible explanation to a report in which 2 circular manic-depressive subjects whose circadian clocks were deemed "too slow" were lithium non-responders, whereas 5 subjects in the same study with a "fast" circadian rhythm free run were responsive [65].

Atack [66] appropriately proposed that any hypothesis on the therapeutic actions of lithium should be able to explain how it controls both mania and depression by modulating the activity of distinct neurotransmitter systems that control these extremes of mood. Direct signaling targets of GSK3 are unlikely to fully satisfy this requirement. However, the modulatory role of GSK3 in the circadian clock mechanism, whose output signals regulate various neurotransmitter and neuropeptide systems, provides the basis for lithium-mediated control of a broad array of neuronal signaling pathways in which abnormalities have been demonstrated in BPD. Moreover, neuroactive drugs do not commonly alter circadian timing [67]. It is therefore highly significant that three other structurally unrelated antidepressants – the second generation monoamine oxidase inhibitor (MAOI) clorgyline, the tricyclic antidepressant imipramine, and the selective serotonin reuptake inhibitor (SSRI) fluoxetine, have been attributed chronobiotic properties in studies of their chronic administration in rodents [6,68].

Considering the subset of BPD patients who are non-responsive to lithium, and the fact that circadian abnormalities do not necessarily result in mood disorders [6], and vice versa, the inhibition of GSK3 by lithium in the context of circadian rhythms is hardly a panacea for the therapy of affective mood disorders. Nevertheless, (i) the cyclical and episodic nature of BPD symptoms, (ii) the prevalence of circadian rhythm abnormalities in BPD, (iii) the consistent evolutionarily conserved period/phase altering effects of lithium, (iv) the direct inhibition of GSK3 by lithium in vivo and in vitro, and (v) the role of *Sgg/GSK3 *in the modulation of period length in fruit fly and murine clocks, places GSK3 at the forefront of the short list of known physiological targets in the therapeutic efficacy of lithium in BPD.

## Competing interests

The author(s) declare that they have no competing interests.

## Authors' contributions

**SK **carried out the serum shock culture assays, RNA isolation of samples, PCR amplification and Western analysis of samples, participated in the design of the study, and drafted the manuscript. **BD **generated the RNAi GSK3 mutant lines (A1.4 and A1.6), generated the c2.1 3/4 DKO mutant, and carried the clonal Western analysis and selection. **NA **participated in the analysis of results and figure designs. **JW **participated in the design of the study. **AM **conceived of the study, participated in its design and coordination, and helped to draft the manuscript. All authors read and approved the final manuscript.
